# Benefits from one session of deep and slow breathing on vagal tone and anxiety in young and older adults

**DOI:** 10.1038/s41598-021-98736-9

**Published:** 2021-09-29

**Authors:** Valentin Magnon, Frédéric Dutheil, Guillaume T. Vallet

**Affiliations:** 1 CNRS UMR 6024, LaPSCo, Université Clermont Auvergne, Clermont-Ferrand, France; 2 School of Exercise Science, Faculty of Health, Australian Catholic University, Melbourne, Australia

**Keywords:** Public health, Therapeutics

## Abstract

Anxiety is recognized as a major health issue and is quite prevalent among older adults. An efficient way to manage anxiety is abdominal breathing. Breathing exercises seem to reduce anxiety and to increase parasympathetic activity assessed by HRV indexes. Yet, the effect of abdominal breathing on physiological stress (HRV) and anxiety in older adults remains poorly understood. Therefore, the aim of this study is to test the effects of deep and slow breathing (DSB, low inhale/exhale ratio) on physiological stress and anxiety in older adults (n = 22) in comparison with younger ones (n = 25). DSB increased significantly HFpower and reduced state anxiety in both younger and older adults. Interestingly, the increased in HF power was significantly higher among older adults than younger ones. As expected, the ratio inhale/exhale being not equal, RMSSD did not increase following DSB. Thus, we provide evidence suggesting that DSB is more beneficial to older adults than younger ones to restore vagal outflow. Despite future work being required, those results provide relevant clinical application leads to manage state anxiety among older adults and to promote successfull aging.

## Introduction

Anxiety and its associated physiological stress response have became ubiquitous in our modern societies^1,2^. Anxiety is one of the most common complaints^3^ frequently associated with poor mental health^4^ at a psychological level and increased all-cause mortality^5^ at a medical level. Anxiety can be defined as a temporally diffused emotional state, in anticipation of a potential threat, associated with physiological stress responses^6^. The consequences of anxiety might even worsen with age as physiological changes reduce the body’s ability to adapt to it. More precisely, healthy older adults exhibit higher mean diurnal levels of stress biomarkers than younger individuals^7^. Short-term physiological stress responses are healthy regulations^8^; however, more prolonged physiological responses to anxiety can lead to an impaired ability to recover from anxiogenic events^9^, increased medical diseases (e.g., increased risk of chronic diseases^10^), cognitive disorders (e.g., memory impairments^11^) and degraded mental health (e.g., depressive symptoms^12^ and notably anxiety disorders^13^). Elderly adults are aware of these effects as anxiety is quite prevalent in this population^14^. These data highlight the need to develop an efficient treatment for anxiety. Even though several pharmacological treatments exist, they are sometimes ineffective and associated with harmful iatrogenic effects^15,16^. By contrast, psychotherapeutic interventions have gained in popularity^17^ and some of them, in particular abdominal breathing, are efficient, cheap and simple ways to reduce anxiety^18^.

Breathing can indeed directly affect the activity of the autonomic nervous system, including the heart rate^19^. Heart rate is regulated by a dynamic balance between the sympathetic nervous system (mainly associated with physiological “*flight or fight*” responses) and parasympathetic nervous system (depending on vagal activity, mainly related to energy conservation, rest, relaxation, etc.^20^). During inhalation, the cardiovascular center inhibits vagal outflow, thus resulting in sympathetic predominance which speeds up the heart rate^21^. Conversely, during exhalation, the vagal outflow is restored and results in a slowing-down of the heart rate^22,23^. The balance between sympathetic and parasympathetic influences is reflected by heart rate variability (HRV), which is the fluctuation of instantaneous heart period over time^24,25^. Since HRV reflects the activity of the autonomic nervous system, it is often used to reliably assess the physiological stress response (as a low parasympathetic activity is considered as a marker of stress)^26^ and can be a powerful tool for testing the effect of relaxation techniques based on breathing. The high frequency (HF) of the HRV power spectrum coincides with respiration (typically 0.15 to 0.4 Hz in adults) under conditions of parasympathetic activity^27^, while lower frequencies (LF, 0.04 to 0.15 Hz) seem to be associated with sympathetic activity^28^, although this is still a subject of debate. Therefore, a breathing exercise (even as short as 5 min) proposing an equal inhalation/exhalation ratio^29^ promotes a balance between sympathetic and parasympathetic activity^30,31^, increases HRV and promotes psychophysiological coherence (for a review on the link between HRV and self-regulation, see^18^).

In the case of anxiety, it might be preferable to reinforce the parasympathetic activity beyond the point of equilibrium in order to maximize relaxation^32^. This aim could be achieved by using deep and slow breathing (DSB), a method characterized by a longer exhale than inhale duration. Accordingly, 5 min of DSB efficiently increases parasympathetic activity, as assessed by HF power (i.e., % of HF in the total HRV power) and decreased perceived anxiety level among young adults^33–35^. Therefore, DSB should constitute a reliable method for promoting vagal tone and reducing anxiety. Nevertheless, and surprisingly, no study to date has tested the effects of DSB (i.e., exhalation longer than inhalation) on physiological stress and perceived state anxiety among older adults. Even though aging is associated with endothelial dysfunction and arterial stiffness^36^ and reduced autonomic reactivity^37^, vagal modulation of the heart rate appears preserved^38^. As a consequence, older adults should still benefit from DSB, albeit to a lesser extent than younger adults.

### Hypotheses

The purpose of this study was therefore to compare the effects of a 5-min DSB exercise (pre vs post-DSB) on subjective (i.e., self-reported) state anxiety and physiological stress (i.e., measured by HRV) in young and older adults. It was expected that anxiety should decrease following the DSB exercise. Moreover, DSB should specifically increase HF power (and not another parasympathetic HRV index less affected by respiration), as exhalation is longer than inhalation, among both young and older adults. However, this increase should be greater in young than in older adults due to their healthier cardiovascular systems. Finally, state anxiety should be negatively correlated with HF power.

## Methods

The experimental protocols were approved by the French South-West and Overseas Regional Ethics Committee for Medical and Health Research Ethics (ID-RCB: 2020-A02193-36). The procedures were carried out in accordance with the approved recommended guidelines for assessing HRV variables in psychophysiological research^39^. Informed consent was obtained from all participants before the experimental session started. Sample size was determined by using the “*pwr*”^40^ package in R with the parameters found in Chinagudi et al. (2014)^41^. The data obtained from the sample made it possible to compute an effect size of *Cohen’s d = .85.* which was associated with a within-study design, with .95 power and .05 as significance level. A sample size of 20 participants per group was then determined as the minimum necessary to conduct the study.

### Participants

In total, 71 participants (including 30 older adults and 41 young adults) were enrolled in this study. In exchange for their participation, participants could win one of four gift vouchers worth 50 each. Participants were eligible to participate if they did not present prior cardiovascular illnesses (e.g. arrhythmia, heart failure), severe inflammation (e.g. arthritis), or a neurological (head trauma, epilepsy, etc.), physiological (hypo- or hyperthyroidism, type 2 diabetes) or neurodevelopmental disorder (autism, dyslexia, etc.). Furthermore, they did not take any medication influencing the cardiovascular system (e.g. antidepressant, antipsychotic, antihypertensive, psychotropic) and they met certain sociodemographic criteria (age, sex, fluency in French etc.). Beyond medical history, lifestyle habits which might affect the functioning of the autonomic nervous system were also collected. These included smoking, drinking alcohol or the body mass index (BMI). Self-reported sleep quality and quantity were also controlled. The Hospital and Anxiety Depression Scale (HADS^42^) and the Mini Mental State Examination (MMSE^43^) were administered to measure anxiety, depression and global cognitive functioning, respectively. Following the test norms, a cut-off score $\ge$ 11 was used as a clinical score for the anxiety and depression subscales of the HADS, and a cut-off of < 27 was used for the MMSE. 8 participants were excluded based on these criteria. Among the young participants, 5 had a high depression or anxiety score and 1 had a BMI of > 35. One older adult had an elevated anxiety score and one had a score < 27 on the MMSE. For 16 more participants, there was a technical issue affecting physiological data acquisition which prevented us from analyzing the data. The final sample thus consisted of 47 participants, namely 25 young and 22 older adults (see Table 1).

**Table 1:** Sample descriptive data.

	Overall	Young adults	Older adults	*p*
n	47	25	22	
Age (mean (SD))	41.23 (23.63)	19.56 (1.64)	65.86 (5.05)	<0.001
Sex = M (%)	9 (19.1)	4 (16.0)	5 (22.7)	0.831
MMSE (mean (SD))	27.94 (1.41)	27.76 (1.39)	28.14 (1.42)	0.365
HAD$_{Dep}$ (mean (SD))	3.04 (2.04)	3.36 (2.18)	2.68 (1.86)	0.261
HAD$_{Anx}$ (mean (SD))	7.21 (3.51)	7.92 (3.40)	6.41 (3.54)	0.143
SleepH (mean (SD))	7.47 (1.33)	7.44 (1.40)	7.50 (1.28)	0.886
SleepQ (mean (SD))	6.03 (2.44)	5.53 (2.47)	6.60 (2.32)	0.131

MMSE, Mini Mental State Examination; HAD Dep, Hospital Anxiety Scale, Depression subscale; HAD Anx, Hospital Anxiety Scale, Anxiety subscale; SleepH, Hours of sleep; SleepQ, Sleep quality.

### Material

#### Perceived state anxiety

Spielberger’s State Anxiety Inventory (SAI) scale^44^ is a self-reported 20-item questionnaire assessing anxiety. The questionnaire focuses on the current state of anxiety, asking how participants feel “right now,” in response to items that measure subjective feelings of apprehension, tension, nervousness, worry, and activation/arousal of the autonomic nervous system.

#### Physiological stress

Stress response was assessed using the EMPATICA E4 Wristband (Empatica E4, Italy), which is a medical-grade wearable device that permits the acquisition of physiological data such as electrodermal activity, temperature and heart rate. The reliability and accuracy of EMPATICA E4 for research on HR and HRV have been previously demonstrated through comparisons with electrocardiography (ECG) measurements^45^. Heart rate was recorded in order to extract the inter-beat intervals (IBI). IBI were then used to obtain HRV outcomes using the Kubios HRV software (v.3.3.1). A visual check was performed to identify the presence of artefacts or occasional ectopic beats and a very low threshold filter was applied when judged necessary.

The two main HRV outcomes were the High Frequency band (HF) and the *Root Mean Squared of Successive Differences* (RMSSD) as they reflect parasympathetic activity through parasympathetic mediated changes in HRV^46^. The HF spectrum is a frequency domain measure (range from 0.15 to 0.4 Hz), called the *respiratory* band, that reflects parasympathetic or vagal activity on variations affecting the respiratory circle^47^. The HF power was computed in normal units as a percentage of the normalized total power which ranges from 0.04 to 0.4 Hz (total power minus the very low frequency)^48^. The remaining percent are therefore LF, which will not be directly taken into account in this study given that the interpretation of LF and thus of the LF/HF ratio is controversial^28,47,49^. The RMSSD is a time-domain measure obtained by calculating all the successive time differences between heartbeats, which are then squared and averaged before the squared root of the total is obtained^47^. As such, it reflects the beat-to-beat variance and therefore the fitness of the heart to adapt itself to sudden external or internal pressures. In order to specify the effect of DSB, its effects on RMSSD were also tested. While being correlated with HF^50^, RMSSD seems to be less affected by respiration^51,52^. Thus, no significant differences in RMSSD following DSB were expected among either young or older adults. The physiological data were recorded for the whole session in order to accustom participants to wearing the wristband. By placing tags (or triggers) on the recording, the pre-test and post test periods were timed to last between three and 5 min (the recommended range for assessing short-term HRV^53^) and took place, respectively, immediately before and after DSB, with the participants comfortably seated and not engaged in any cognitive demanding task.

#### Deep and slow breathing

The breathing exercise was guided by a video displayed on a computer screen on which a drop of water moved up and down in circles. When the drop went up, the participant had to inhale and when the drop went down the participant had to exhale. At first, the inhalation and exhalation duration were equal (4 seconds) and then, little by little, exhalation became longer than inhalation (4 seconds in and 6 seconds out). The whole exercise lasted 5 min.

### Procedure

This study was part of a larger project with multiple objectives. As such, several of the tests that the participants underwent are not taken into account in this study. Each participant was tested individually in a session lasting approximately 1 hour. Firstly, the experimenter fitted the Empatica wristband on the participant’s left wrist and turned the device on. The participant then completed the health and sociodemographic interview and inclusion/exclusion test (HADS and MMSE). Next, a 10-min computerized task was administrated (either an emotional categorization task or a false recognition task). After this, a trigger was set by quickly pressing the wristband switch to start recording the pre-test period during which the participants completed the state anxiety questionnaire (SAI). A new trigger was set to mark the end of pre-test and the beginning of the induction. The DSB exercise was then completed. A third trigger indicated the start of the post-test and the participant completed the state anxiety questionnaire (SAI) a second time. The fourth trigger marked the end of the post-test. Finally, the participant completed an interoception test (heart beat detection task^54^), *échelle d’anxiété évaluation état* (EAEE^55^), the second computerized task (counterbalanced with the first one described above), the emotion regulation questionnaire (ERQ^56,57^), the French National Reading Test (fNART^58^), the HADS^42^ and the White Bear Suppression Scale Inventory (WBSI^59,60^). Specifically, the order of the computerized tasks was counterbalanced so that half the participants undertook the emotional categorization task first (before the pre-test) and then the false recognition task (after the post-test) while the other half undertook the tasks in reverse order (first the false recognition task then the emotional categorization task). The session ended with a debriefing on the experiment.

### Statistical analyses

All the statistical analyses were performed using RStudio software^61^. For all statistical analyses, the employed statistical significance threshold was set at *p* < 0.05. Pre-DSB and post-DSB anxiety scores were obtained from the SAI questionnaire. Since these variables were very left-skewed, the data did not meet the normality assumption. Therefore, Wilcoxon tests (non-parametric tests) were conducted to determine whether the reduction in self-reported anxiety occurred after DSB across all participants and within each age group. RMSSD and HF power were obtained from the HRV analyses performed with Kubios on the data from the Empatica E4 Wristband. A visual examination confirmed the normality of the distribution. A mixed analysis of variance (ANOVA) using a 2x (Group: young *vs* older participants) by 2x (Time : pre DSB *vs* post DSB) design was therefore conducted, with Group as between-subject variable and Time as within-subject variable. Post-hoc analyses were conducted to examine the interaction between the effect of DSB (Time: pre *vs* post) and age (Group: young *vs* older). More precisely, the differences between young and older adults in the pre- and post-DSB were investigated using the Tukey procedure (Bonferroni correction for multiple comparisons). A correlation analysis was performed to investigate the association between post-DSB anxiety scores and post-HF. As the post-DSB anxiety scores did not meet the normality assumption, the correlation test was computed using the $\rho$Spearman method (non-parametric test). The Spearman correlation method is known to be resistant to outliers, making it a robust and effective way to conduct correlations on variables that deviate from normality^62^.

## Results

### Perceived state anxiety

A Wilcoxon test indicated that the anxiety level was lower after ($M_{post} =23.44, SD_{post} = 4.37$) the DSB exercise than before ($M_{pre}~=~26.95, SD_{pre} = 6.12$) among all participants ($W = 2,749.50$, $p < .001$). This difference was observed in both young ($M_{pre} =28.60, SD_{pre} = 6.49$ *vs.* $M_{post} =24.46, SD_{post} = 4.88$), $W = 866.50$, $p = .003$, and elderly adults ($M_{pre}~=~24.89, SD_{pre} = 5.01$ *vs.* $M_{post} =22.18, SD_{post} = 3.29$), $W = 531.00$, $p = .019$.

### Physiological stress

**Figure 1:**
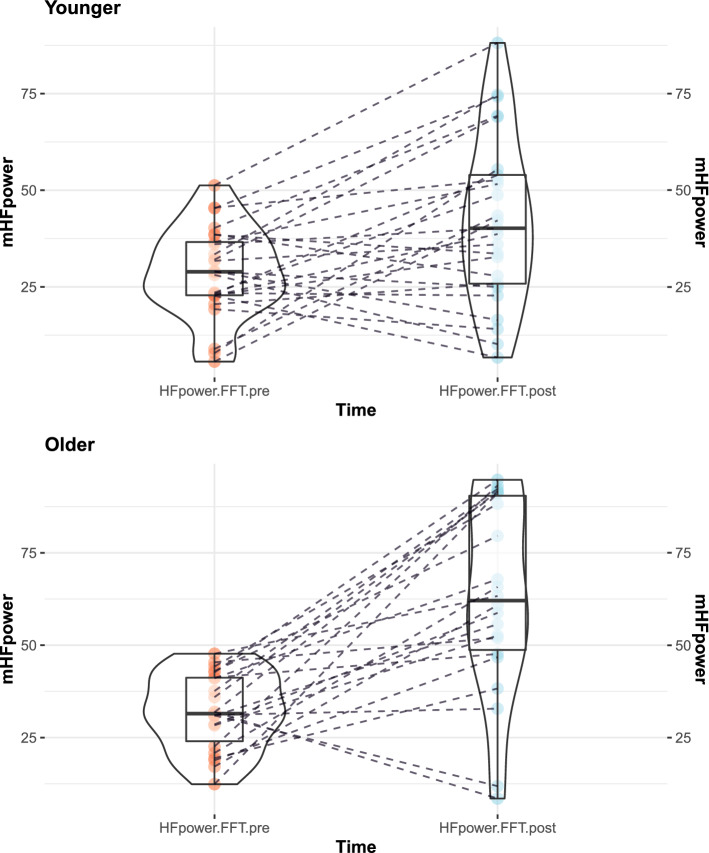
Effect of the interaction between time and age on HFpower.

**Figure 2:**
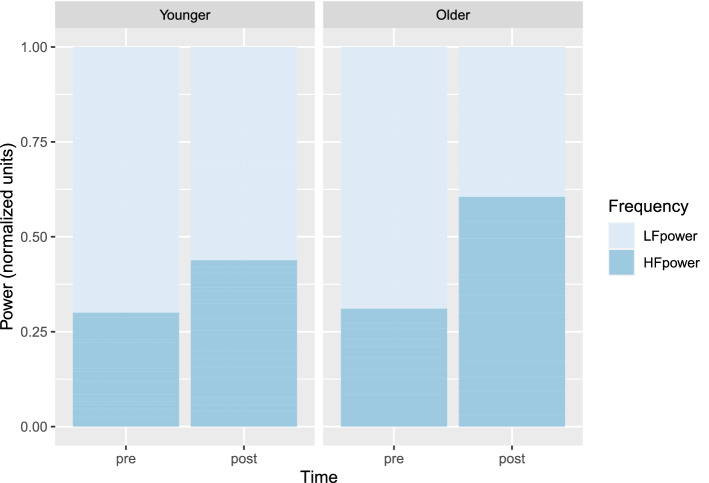
HF and LF repartition according to time and age.

**Figure 3:**
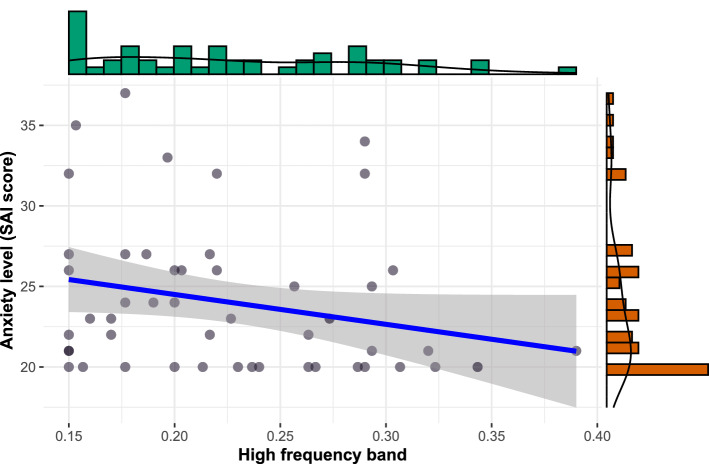
Negative association between HF and post-DSB anxiety scores.

The ANOVA performed on HF power (see Fig. 1) revealed a main effect of Time ($F(1, 45) = 42.64$, $\textit{MSE} = 271.17$, $p < .001$, ${\hat{\eta }}^2_G = .273$), a main effect of Group ($F(1, 45) = 8.26$, $\textit{MSE} = 413.51$, $p = .006$, ${\hat{\eta }}^2_G = .100$) and a significant one-way interaction ($F(1, 45) = 6.58$, $\textit{MSE} = 271.17$, $p = .014$, ${\hat{\eta }}^2_G = .055$). The increase in HF power among older adults ($M_{pre} = 31.01, SD_{pre} = 10.81$ *vs.* $M_{post} = 60.33, SD_{post} = 28.20$) appeared to be significantly higher than that among young adults ($M_{pre} = 29.97, SD_{pre} = 13.39$ *vs.* $M_{post} = 43.72, SD_{post} = 21.53$). Posthoc analyses revealed no significant difference between younger adults ($M_{pre} = 29.97, SD_{pre} = 13.39$) and older ones ($M_{pre} = 31.01, SD_{pre} = 10.81$) in pre-test HF power ($\Delta M = -1.04$, 95% CI $[-7.72, 5.64]$, $t(53) = -0.31$, $p = .756$), whereas a significant difference was revealed at post-test ($\Delta M = -16.62$, 95% CI $[-30.62, -2.62]$, $t(49) = -2.39$, $p = .021$) showing a higher HFpower among older ($M_{post} = 60.33, SD_{post} = 28.20$) than younger adults ($M_{post} = 43.72, SD_{post} = 21.53$). Figure 2 provides a comparison between HF power and LF power (remaining percent of total power) according to Time and Group.

In order to test whether physiological stress is associated with state anxiety, a correlation between HF band and anxiety score was calculated (see Fig. 3). The HF band (Hz) was negatively associated with anxiety score after DSB ($r_{\mathrm {s}} = -.29$, $S = 28,427.46$, $p = .042$) suggesting that the higher HF is, the lower the anxiety level.

In order to specify the effect of DSB, the RMSSD (another parasympathetic HRV index which is reportedly less affected by respiration) was also tested (for a summary of descriptive data, see Table 2). An ANOVA showed no main effect of Group ($F(1, 46) = 2.56$, $\textit{MSE} = 691.77$, $p = .116$, ${\hat{\eta }}^2_G = .040$), no significant main effect of Time ($F(1, 46) = 0.34$, $\textit{MSE} = 244.28$, $p = .563$, ${\hat{\eta }}^2_G = .002$) nor any interaction effect ($F(1, 46) = 2.98$, $\textit{MSE} = 244.28$, $p = .091$, ${\hat{\eta }}^2_G = .017$) on RMSSD.

**Table 2:** Mean (SD) for HF power and RMSSD across the young and older adult groups in the pre- and post-test.

Time	Young adults		Older Adults	
	Pre	Post	Pre	Post
HF power	29.97 (13.39)	43.72 (21.53)	31.01 (10.81)	60.33 (28.20)
RMSSD	61.29 (23.71)	55.75 (15.90)	65.10 (20.85)	66.38 (26.10)

## Discussion

The aim of the present study was to assess the effect of a DSB exercise on both perceived state anxiety and physiological stress (HF-HRV) in older compared to young adults. We hypothesized that DSB would reduce the perceived anxiety level in both groups and decrease physiological stress more in the young than the older adults. Regarding HRV, it was expected that, specifically, HF power (reflecting parasympathetic activity and corresponding to the HR variations related to the respiratory cycle) would increase following the DSB exercise (the inhalation/exhalation ratio being low). By contrast, no difference was expected on the RMSSD, because it should be less affected by respiration.

As expected, subjective anxiety, significantly decreased among both young and older adults after only a 5-min DSB exercise. In addition, physiological stress also decreased after DSB, as indicated by a significant increase in HF power among younger and older adults. These results are consistent with the small number of other studies that have investigated the efficacy of DSB on vagal activity and on perceived anxiety level (e.g.,^33–35^ among young adults). For instance, one study compared DSB (low inhalation/exhalation ratio) and breathing with a high inhalation/exhalation ratio on HRV among undergraduate students and found that only the former specifically increased HF power and induced a relaxation state^35^. DSB has, surprisingly, never been studied among older adults. However, a few studies have shown that resonant breathing increases HRV indexes such as the RMSSD^63,64^. Nonetheless, RMSSD is probably not the best index of parasympathetic functioning because its calculation takes into account beat-to-beat variance and may thus reflect both parasympathetic and sympathetic activities^65^. Therefore, RMSSD should be more likely to be affected by resonant breathing (equal inhalation/exhalation ratio), which promotes a balance between the two branches of the autonomic nervous system, whereas DSB should more specifically target HF. Furthermore, RMSSD appears less susceptible to variations in respiratory frequency^51,66^. Therefore, it might be expected that RMSSD would not change following DSB in young or older adults as was observed in the present study.

The effects of DSB on the physiological and psychological state of the individual can be explained by psychophysiological models such as the polyvagal theory^32^. According to this theory, the autonomic nervous system evolved in order to influence cognitive information processing in response to contextual cues. Sympathetic activity is thought to be linked to stress responses that would trigger hypervigilance and anxiety. Conversely, parasympathetic activity, occurring in secure environments, would instead be associated with social cognition and emotion regulation in order to promote efficient cooperation that increases the odds of survival^32^. This theory predicts that a low inhale/exhale ratio should specifically increase vagal activity and not sympato-vagal balance, resulting in a relaxation state that promotes efficient social interactions. This is congruent with the increased HF power, the reduced state anxiety and the negative association between anxiety and HF band observed in the present study. Taken together, these empirical and theoretical data suggest that influencing vagal outflows by means of DSB is an efficient way to reduce anxiety level.

Our results go a step further by testing the effects of DSB on older adults in comparison to young ones. Unexpectedly, the increase in HF power was significantly greater in the older adults than in their younger counterparts. This difference occurred even though the participants in both groups showed an equivalent level of HF power at baseline, whereas, after DSB, the HF power level was significantly higher among older adults than younger ones. In other words, DSB seems to benefit vagal outflow more in older participants. This finding is congruent with studies investigating transcutaneous vagus nerve stimulation that have suggested that vagal stimulation could be particularly effective in healthy older compared to younger participants^67,68^. Such results might illustrate a greater benefit in terms of vagal tone increase in line with theories suggesting that older adults have enhanced emotion regulation skills^69^ which are mainly influenced by parasympathetic indexes such as HF^70,71^. With age, vagal modulations of heart rate should be maintained^72^ and parasympathetic HRV indexes follow a U-shaped curve with a reversal increase above 60 years old^73,74^. Similarly, studies have suggested that HRV is a marker of healthy aging associated with stress management among older individuals and that age-related decline in HRV is not inevitable^75^. By contrast, the HF band provides an index of psychological resilience, behavioral flexibility and the capacity to adapt to changing social demands^76^. As such, DSB, by promoting vagal nerve activity through a low inhale/exhale ratio, could be a catalyst for optimal anxiety management and emotion regulation. If this is indeed the case then a longer induction might not only greatly increase vagal tone, but it may also decrease state anxiety more greatly among older adults. The present results still report a similar reduction in anxiety level in the two age groups.

Another explanation may therefore be that greater vagal activation in older adults is required in order to effectively reduce perceived anxiety, in the same way as has been observed in younger individuals. Neuroimaging studies have revealed a similar mechanism, with greater activation (fMRI, functional magnetic resonance imaging) being observed in frontal regions in older people in order to achieve equivalent memory performances to those of younger adults^77,78^. In the same way, there might be an over-investment of vagal activity at ages over 60 years^74^ in order to counterbalance the age-related reduction in resources, for example by increasing the level of stress biomarkers^7^ to cope with anxiogenic situations^9^. If this is the case, the increase in the benefit brought about by the greater increase would not be specific to older adults, but rather an age-related compensation mechanism that maintains anxiety management with age.

Despite the positive nature of the outcomes, the current study is not without limitations. Even though the total number of participants was adequate according to our pre-hoc power calculation, the results obtained in our male and female participants were analyzed together (the proportion of males in both age groups being equivalent). Nevertheless, a sexual dimorphism seems to influence autonomic functioning^79^ and its response to stress^80^. Moreover, the breathing exercise used in the present study was not individually adapted based on the spontaneous respiration of each participant^81^. We can nonetheless state that no age difference was found in the normal respiratory rate^82^. Even though we controlled for sleep quality and quantity and BMI, there are also potential confounds that may contribute to the present results, such as the practice of physical activity^83^, yoga^84^ or meditation^85^ and other lifestyle factors that are likely to influence HRV^86^,such as sedentariness^87^, a factor that is independent of physical inactivity^88^. Finally, there was no control group, which makes it difficult to rule out other possible explanations for the results obtained in the present study, such as test-retest effect despite the good psychometric properties of the SAI and physiological measures. Future studies addressing these limitations would be necessary to refine the conclusions drawn from these initial results.

Nonetheless, this study provides evidence that: 1) DSB seems to reduce anxiety level and increase vagal outflow (which are negatively correlated with each other) in both young and older adults; 2) DSB could have a greater effect on parasympathetic activity in older adults. This greater effect could reflect a greater benefit of vagal tone contributing to better emotional regulation strategies with age or possibly a compensation mechanism promoting efficient anxiety management. Whatever the case may be, these results suggest that DSB might be a useful and efficient intervention for acute anxiety management among young adults and, to an even greater extent, among older adults. Beyond acute anxiety, DSB may also constitute an interesting method for preventing the deleterious effects of long-term anxiety effects on health^5^ or might even contribute to successful aging. Indeed, the neuro-visceral integration^89^ and the psycho-physiological coherence model^18^ indicate that higher HRV (particularly HF band) is associated with better cognitive performance^90^. Higher vagal activity also predicts better emotion regulation strategies, such as avoidance of the negativity and positivity effect in aging^91^. As such, DSB represents a practical, low-cost exercise that can be performed anywhere in order to promote successful aging. Finally, to adopt a more clinical perspective, DSB would fit very well among the efficient techniques offered as part of Acceptance and Commitment Therapy (ACT) in order to reduce anxiety, stress, or pain^92^ in older adults, among other individuals^93^.

## Supplementary information

Supplementary Information.
